# Temporal changes in abundance–occupancy relationships over 40 years

**DOI:** 10.1002/ece3.5505

**Published:** 2019-09-30

**Authors:** Lisa L. Manne, Richard R. Veit

**Affiliations:** ^1^ Biology Department College of Staten Island City University of New York Staten Island New York; ^2^ Biology Doctoral Program (EEB) CUNY Graduate Center New York New York

**Keywords:** abundance–occupancy, avian ecology, breeding bird survey, geographic range, macroecology, migratory guild, population monitoring, temporal change

## Abstract

1. Abundance–occupancy (A–O) relationships are widely documented for many organismal groups and regions, and have been used to gain an understanding of regional population and community trends. Monitoring changes in abundance and occupancy over time may be what is required to document changes in conservation status and needs for some species, communities, or areas.
2. We hypothesize that if there is a higher proportion of declining species in one group of species compared with another (e.g., migratory species vs. permanent residents), then a consequence of that difference will be vastly different abundance–occupancy relationships. If this difference persists through time, then the resulting A–O relationships between the groups will continue to diverge.
3. For neotropical migrants, short‐distance migrants, and permanent resident birds of North America, we assess the numbers of declining species over 1969–2009. We further test for differences in the A–O relationship across these three groups, and in rates of change in abundance and occupancy separately.
4. We find significant differences in numbers of declining species across the migratory groups, a significant decline in the A–O relationship for permanent residents, a significant increase for Neotropical migrants, and a nonsignificant decline for short‐distance migrants over the 40 years. Further, abundances are not changing at different rates but occupancies are consistently greater over time for neotropical migrants versus permanent residents, likely driving the changes in A–O relationships observed.
5. In these analyses, we documented changing A–O trends for different groups of species, over a relatively long time period for ecological studies, one of only a few studies to examine A–O relationships over time. Further, we have shown that a temporally unvarying abundance–occupancy relationship is not universal, and we posit that variability in A–O relationships is due to human impacts on habitats, coupled with variation in species' abilities to respond to human impacts.

## Graphical abstract

Under current circumstances of habitat loss and degradation, macroecological relationships are changing. In this manuscript, we documented changing abundance–occupancy trends for different groups of bird species, over a 40‐year time period. We further showed that a temporally unvarying abundance–occupancy relationship is not universal, and we posit that variability in A–O relationships is due to human impacts on habitats, coupled with variation in species' abilities to respond to human impacts.

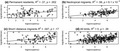

## 1 INTRODUCTION

Interspecific abundance–occupancy (hereafter abundance–occupancy or A–O, unless qualified by “intraspecific”) relationships are widely documented for many organismal groups and regions (Blackburn, Cassey, & Gaston, 2006); Darwin theorized their occurrence (Darwin, 1859; Zuckerberg, Porter, & Corwin, 2009). Abundance–occupancy (A–O) relationships have been used to gain an understanding of regional population and community trends (Donald & Fuller, 1998; Freckleton, Noble, & Webb, 2006; Gibbons, Donald, Bauer, Fornasari, & Dawson, 2007). A–O relationships are particularly useful because declining abundance is not always accompanied by declining range (Böhning‐Gaese & Bauer, 1996; Chamberlain, Fuller, Bunce, Duckworth, & Shrubb, 2001). Having the information provided by both occupancy and abundance, and monitoring changes in both over time may be what is required to rapidly document changes in conservation status for some species and to gauge community disruption in a timely manner (Ormond, Whatmough, Hudson, & Daniels, 2014).

The interspecific A–O relationship is typically positive: As species occupy more geographic area, they tend to have higher average abundances across the range. There are several reasons for why this positive relationship can be observed in nature: resource use dynamics, range position, sampling artifact, and resource availability. Species with different niche breadths (and different patterns of resource use) will achieve different areas of occupancy and different average abundances across their ranges. Aggregating across all species will yield a positive abundance–occupancy relationship (Gaston & Lawton, 1994; Warren & Gaston, 1997). Range position within the study area will result in a positive relationship, because species that occupy most or all of the study area will have higher average abundances than other species (Warren & Gaston, 1997). Similar sampling artifacts might arise through incomplete sampling of species with the smallest ranges and/or abundances. Within the study area, species utilizing abundant resources will themselves achieve high abundances, and species utilizing rare resources will be correspondingly less abundant. See a very good review of drivers in Warren and Gaston (1997) and of performance of A–O models at predicting abundance from occupancy (Hui et al., 2009). The latter paper makes the excellent point that the interspecific A–O relationship will be constrained by the intraspecific relationships of its composite species and that (at least for the data examined by Hui et al. (2009)) abundance can be reliably estimated from occupancy at finer scales, but will be underestimated at larger scales.

Negative interspecific A–O and/or no relationships have also been observed (Blackburn et al., 2006; Chamberlain et al., 2001; Päivinen et al., 2005; Symonds & Johnson, 2006). Speculations about causes of negative (or a lack of: Reif et al., 2006) relationship between abundance and occupancy include effects of isolation coupled with a high degree of adaptation to the environment (Reif et al., 2006), range position (Päivinen et al., 2005), and differences in niche breadth resulting in a negative relationship between niche breadth and density (Päivinen et al., 2005). Thus far, these negative relationships between occupancy and abundance are rare; they deserve further study, to understand why they might arise. The number of documented positive A–O relationships far exceeds the number of negative relationships.

For abundance–occupancy relations to provide a reliable barometer of community change, they would need to remain stable over time. Few studies have examined the consistency of interspecific abundance–occupancy relationships over long time periods. Three notable exceptions are studies by Fisher and Frank (2004), Webb, Noble, and Freckleton (2007), and Zuckerberg et al. (2009). Fisher and Frank (2004) studied marine fish species under pressure from fishing. The community showed a decreasing abundance–occupancy trend over time for the 32 years studied. The authors note that both occupancy and abundance of individual species changed in response to the fishing activity, thus driving the change in the abundance–occupancy relationship over the study period. Webb et al. (2007) assessed avian abundance–occupancy dynamics in Great Britain over 32 years, contrasting these in farmland and woodland environments. They found that the strength (measured as correlation coefficient) of the interspecific relationship between abundance and occupancy declined over the study period. They ascribe this declining strength to a weakening of the linkage between abundance and occupancy for the rare species *intraspecific* abundance–occupancy relationship, likely due to a decline in habitat quality. They found that common species’ intraspecific abundance–occupancy relationships resembled the interspecific pattern. Last, Zuckerberg et al. (2009) examined possible temporal dynamics of the interspecific abundance–occupancy relationship for breeding birds in New York state, using two independent data sources. They found that within New York, the A–O relationship was consistent across time, and unvarying across migratory groups and different habitats.

These three studies have examined the possibility in changing A–O relationships over time and have found conflicting results. Both Fisher and Frank (2004) and Webb et al. (2007) cite human‐induced habitat change as major drivers of change in A–O relationships. To reconcile the Zuckerberg study, it is probable that the habitats in New York state have not degraded enough over the time period of that analysis in order to substantially change the A–O relationships (that analysis compares two periods: 1980–5 and 2000–5).

Many species of long‐distance migrants are experiencing rapid and/or sustained population declines across their breeding ranges (Robbins, Sauer, Greenberg, & Droege, 1989; Sanderson, Donald, Pain, Burfield, & Bommel, 2006). Habitat loss and degradation in both wintering and breeding areas are implicated for these declines (Sanderson et al., 2006; Sauer & Droege, 1992; Sherry & Holmes, 1996). In fact, some authors have suggested that avian mortality is sufficiently high during periods of migration to adversely affect breeding population sizes (Klaassen et al., 2014; Newton, 2006, 2007; Sillet & Holmes, 2002; Strandberg, Klaassen, Hake, & Alerstam, 2009), an understudied phenomenon.

Conversely, long‐distance migrants should have more strongly positive A–O relationships because, by virtue of their mobility, they ought to be able to locate and colonize suitable patches of habitat much more readily than short‐distance migrants or permanent residents. Thus, short‐distance migrants and/or permanent residents should be less efficient at occupying habitats, even if their abundances are increasing.

In this analysis, we hypothesize that if there is a higher proportion of declining species in one group of species compared to another (e.g., migratory species vs. permanent residents), then a consequence of that difference will be a vastly different abundance–occupancy relationship between the two groups. Moreover, if this difference persists through time, then the resulting abundance–occupancy relationships between the groups will continue to diverge. For neotropical (long‐distance) migrants, short‐distance migrants, and permanent residents of North America, we assess the numbers of declining species (in abundance, in occupancy, and in number of detections per sampling episode) in the years 1969–2009 and find significant differences in numbers of declining species across the groups of bird species, which then lead to differences in the abundance–occupancy relationship across these three groups.

## 2 METHODS

### 2.1 Data

We use the Breeding Bird Survey dataset, spanning 1969–2009. The North American Breed Bird Survey (BBS) is a yearly transect (“route”) census of North American breeding birds, in operation since 1966, and conducted in early June (or in late May for more southerly routes). The timing is meant to ensure that all migratory bird species have set up breeding territories in their breeding grounds, but that breeding is not very far along so that individual birds are more easily detected by ear (contrasted to later in the season, when birds sitting on nests remain very quiet). Data collected in the BBS are edited so that detections of migratory or nonbreeding species are removed from the dataset (Sauer, Link, Fallon, Pardieck, & Ziolkowski, 2013), and nocturnal species (e.g., a calling owl) are removed, to mitigate possible bias resulting from these species. Each route is 24.5 miles long; there are 50 “stops” along the route, every half mile. Each stop is 3 min long, and census takers record every individual detected (by sight or ear). Observer effort is standardized: There is typically 1 observer per route, per the BBS methodology (Sauer et al., 2013). BBS route locations are chosen so as to allow a representative sample of habitats available within the BBS study region. Route locations are also nonrandom in that they are placed along secondary roads (so as to minimize nondetection due to road noise, and to allow human observers better access to habitat).

### 2.2 Study region

The BBS survey region comprehensively covers the lower half of Canada, the entire US, and Northern Mexico.

### 2.3 Species

We restricted our analyses to terrestrial bird species that breed in the US and Canada, and whose range is largely covered by the BBS survey region. We divided the birds into permanent residents, long‐distance (neotropical) migrants, and short‐distance migrants, following the classification scheme set by the Breeding Bird Survey (Pardieck, Ziolkowski, Hudson, & Campbell, 2016). Permanent residents usually do not migrate to overwintering areas (though many individuals undergo sometimes lengthy migrations); short‐distance migrants do usually migrate, but overwinter within the US and Canada; long‐distance migrants overwinter outside of the US and Canada. These are imperfect designations, as some species that are classified as short‐distance migrants may have portions of the range in which the species is resident, versus portions of the range in which the species migrates (e.g., red‐tailed hawk and American kestrel (Goodrich & Smith, 2008), ospreys (Martell, McMillian, Solensky, & Meale, 2004), many other examples), combined with substantial individual variation within populations.

### 2.4 Occupancy

For each species and for alternate years in the period 1969–2009, we used regression and model selection (Burnham & Anderson, 2002) to assess whether the number of occupied routes (tallied as the number of routes on which a bird was detected in a particular year) statistically increased or decreased (or neither) over time. (A check for temporal autocorrelation of the abundance–occupancy relationship showed significant autocorrelation at a lag of 1 for permanent residents and neotropical migrants (but no autocorrelation for short‐distance migrants), we used alternate years to account for any effects of temporal autocorrelation; this resulted in *N* = 20 years.) We conducted model selection, comparing linear, Poisson and negative binomial models, and selected among those using AIC. In cases where Akaike's information criterion (AIC) was within 4 units of the model with the next‐lowest AIC, we retained the simpler model and tested for statistical significance of the model coefficient(s). Statistically significant model coefficients indicated whether a species has been increasing/decreasing/static in occupancy.

### 2.5 Abundance

Similarly, we used model selection comparing 1st, 2nd, and 3rd order polynomials of average abundance (across all locations where a bird was detected within a particular year) against year, to assess whether a species shows a trend of increasing/decreasing/static population size (number of individuals per route) over time. We used AIC to discriminate among models, retaining simpler models where appropriate (as above for occupancy) and then tested for significance of model coefficients. In cases where the best‐fitting model for abundance was a quadratic model (parabolic in shape), we adopted a decision rule to classify that species’ behavior (for that metric) as the behavior (increasing or decreasing) that was most recent. Since linear and polynomial regression can result in predictions of negative abundance, the usual caveats apply regarding extrapolating beyond the range of the data.

### 2.6 Saturation

We define “saturation” as the proportional number of stops (out of 50) per BBS route on which a species was detected. We used regression to test for increasing, decreasing, or static “saturation” over time. Change in saturation over time indicates whether bird populations are becoming more densely or sparsely distributed within the environments in which they are found. We compared logistic regression with binomial errors (or quasi‐binomial errors for significantly overdispersed data) and used AIC to choose among models, retaining simpler models where appropriate (as for occupancy and abundance) and tested for significance of model coefficients.

We summarized results for these three metrics (occupancy, abundance, and saturation) for the three migratory guilds of bird species (long‐distance migrants, short‐distance migrants, and permanent residents). We tested for significance of coefficients of the best model, and if the coefficients were not significant (i.e., model was “best” according to AIC, but not significant overall), we classified that species as neither increasing nor decreasing for that metric (occupancy/abundance/saturation).

Our goal was to assess changes in species‐specific abundance and occupancy over time, for the three migratory guilds, in comparison with the group comprised of all birds, and to use that information to predict changes in the interspecific abundance–occupancy relationship over time. For us to observe an increasing abundance–occupancy slope over time, the group of species must necessarily have a greater number of species with increasing abundance (compared with the number of species displaying decreased or stable abundance) and/or the species must display decreasing occupancy with stable abundance. Thus, for each migratory guild, we regressed average abundance (the response, taken on a log scale to stabilize the variance) versus log occupancy (number of routes on which a species was detected) over all species in a single year. We retained the slope of each yearly regression and then tested for statistically significant change in slopes of the interspecific abundance–occupancy relationship over time.

## 3 RESULTS

**Figure 1:**
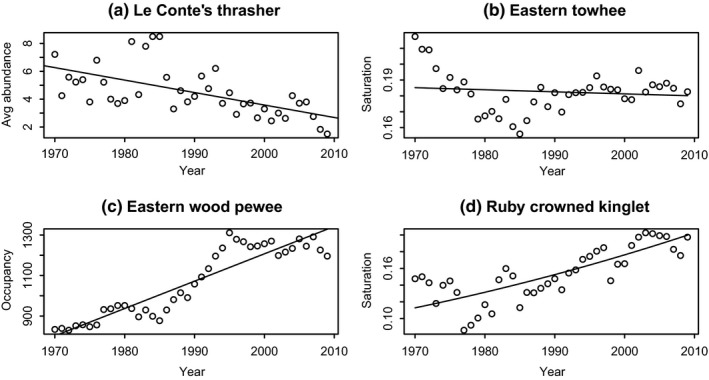
Examples of model results in model selection (a) linear, or for which a linear fit is equally good (as measured by AIC); (b) saturation cannot be said to be increasing or decreasing (nonsignificant logistic model overlaid); (c) negative binomial (gray dashed curve) and linear model (black line) provided equally good fits, so we retained the linear model; (d) this species' saturation increased, as specified by the best model (logistic with quasi‐binomial errors)

For all three metrics (occupancy, abundance, and saturation), there were instances for which the best model was linear (e.g., Figure 1a) and some instances where there was no best (and no significant) model (as Figure 1b). There were additionally some cases where the better model was a polynomial (3rd order) but the overall trend was still clearly increased or decreased (as Figure 1c). In many cases, the best‐fitting model was a curvilinear relationship that closely approximates a line (as Figure 1d, a logistic with quasi‐binomial errors, classified as increasing).

In Table 1, we summarize these results across the three migration guilds, noting proportion of species that show a significantly increasing or decreasing (or no) relationship for occupancy, abundance, and saturation. Over the 40‐year study period, occupancy is increasing on average for all three migratory guilds. For long‐distance migrants, there are a higher proportion of species increasing in abundance (vs. decreasing or static in abundance). Similarly, almost 50% of long‐distance migrants have been increasing in saturation (vs. the 28% that are decreasing in saturation, and the 26% that have shown no statistical change in saturation).

**Table 1:** Proportions of species exhibiting increasing (“inc”), decreasing (“dec”), or no change in Occupancy, Abundance, and Saturation

Migratory guild (# species)	Occupancy			Abundance			Saturation		
	Inc	Dec	No	Inc	Dec	No	Inc	Dec	No
Long distance (138)	0.92	0.01	0.07	0.46	0.28	0.26	0.46	0.28	0.26
Short distance (109)	0.96	0.01	0.03	0.29	0.35	0.35	0.34	0.39	0.37
Permanent residents (108)	0.88	0.01	0.11	0.32	0.45	0.22	0.40	0.26	0.34

The permanent residents show very different results. Permanent residents have a higher proportion of species that are decreasing (in abundance, 45%) compared with those increasing (32%) or static in abundance (22%). Permanent residents have very similar proportions of species increasing and decreasing in saturation (40% and 34%, respectively).

The results for short‐distance migrants' abundance and saturation are similar to those of permanent residents, in that equal proportions of species show declining or static abundance over time (35% each), and very similar proportions of birds are increasing and decreasing in saturation over time (34% vs. 39%, respectively).

Given these results, we checked for significant temporal autocorrelation in the abundance and occupancy relationships (separately for abundance, and then again for occupancy). Temporal autocorrelation does exist, over a period of 3/10/12 years (permanent residents/short‐distance migrants/neotropical migrants). However, this temporal autocorrelation has little bearing on the slope of the abundance–occupancy relationship (see below).

**Figure 2:**
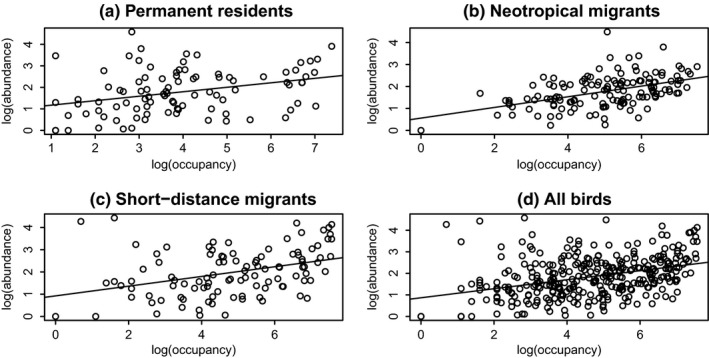
Example (1990) abundance–occupancy relationships

**Figure 3:**
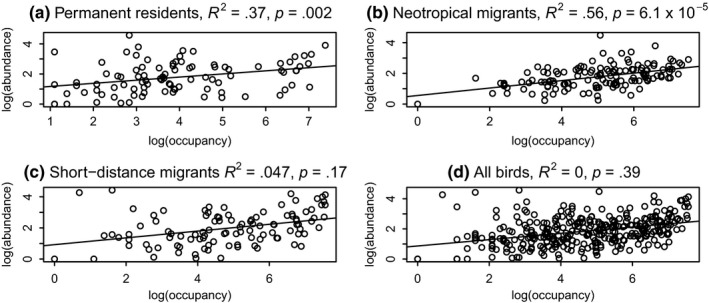
Changes in slopes of abundance–occupancy relationships over the 40 years of this study, for three migratory guilds and for all bird species

Abundance–occupancy relationships are changing over time. Figure 2 shows examples of abundance–occupancy relationships for each migratory guild, for the year 1990 (midway through the study period). Permanent residents showed a statistically significant decrease in slope of the abundance–occupancy relationship over time (*R* ^2^ = .37, *p* = .002, Figure 3a). This means that over the 40‐year period of this study, permanent residents are becoming less abundant in the locations where they are found. Conversely, long‐distance (neotropical) migrants have a statistically significant increase in the slope of the A–O curve over this same period (*R* ^2^ = .56, *p* = 6.1 × 10^–5^, Figure 3b). Short‐distance migrants fall in between, with a nonsignificant decrease in the slope of the A–O relationship over time (Figure 3c). These differences would be masked if all birds were considered in one group (Figure 3d). In this latter case, the observer would conclude that abundance–occupancy relations are not significantly changing over time.

Species with consistently increasing or decreasing abundance might have a biasing effect on our calculation of changes in slopes of abundance–occupancy relationships. However, a test of autocorrelation for the abundance–occupancy relation itself reveals little autocorrelation. For the changing slopes of the abundance–occupancy relationships, and for the three migratory guilds, we assessed the degree of autocorrelation using the pacf command in R. There is significant temporal autocorrelation in the changing abundance–occupancy slopes at a lag of 1 for the neotropical migrants and permanent residents, and no significant temporal autocorrelation for the short‐distance migrants (Appendix S1). Given the significant autocorrelation at a 1‐year time lag, we temporally thinned the data by removing every other year (as in Figure 3) and present analyses and *p*‐values from the thinned data set.

**Figure 4:**
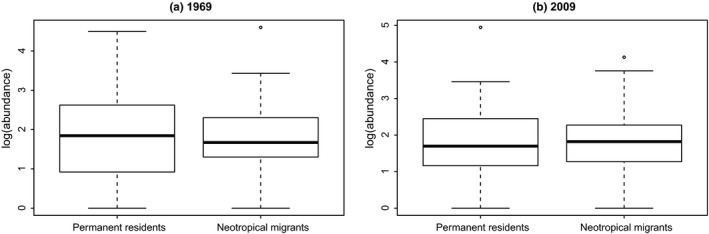
Neotropical migrants and permanent residents have similar abundances in 1969 and 2009 (*t* test, *p* = .57, and .74, respectively)

**Figure 5:**
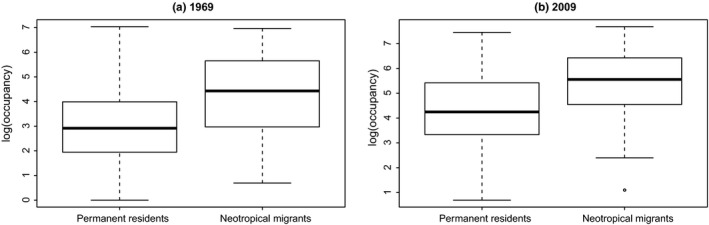
Neotropical migrants have larger occupancies than permanent residents, in 1969 and 2009 (*t* test, *p* = 1.38 × 10^–6^, and 4.03 × 10^–6^, respectively)

To better understand the mechanisms that might be driving the difference in slope of abundance–occupancy relationships across migratory guilds, we assessed changes of occupancy separate from abundance and changes of abundance separate from occupancy. For the 1969 data, there was no significant difference in log‐transformed abundances between the permanent residents and neotropical (long‐distance) migrants (*t* test, *p* = .57, Figure 4); similarly, there was no difference in 2009 abundances between the permanent residents and neotropical migrants (*t* test, *p* = .74, Figure 4). Occupancy (log‐transformed), however, differed between migratory guilds, in both 1969 (*t* test, *p* = 1.38 × 10^–6^, Figure 5) and 2009 (*t* test, *p* = 4.03 × 10^–6^, Figure 5).

Thus, migratory guilds show no difference in abundances over time, but neotropical migrants have larger occupancies over time, and a significantly increasing slope in the A–O relationship, while permanent residents have lower occupancies over time and a significantly decreasing slope in the A–O relationship. These results indicate that in aggregate, neotropical migrants are managing to gain access to more habitat over time than are permanent residents.

## 4 DISCUSSION

We expected that long‐distance migrants would have more strongly positive A–O relationships because, by virtue of their mobility, they ought to be able to locate and colonize suitable patches of habitat more readily than short‐distance migrants. Thus, short‐distance migrants should be less efficient at occupying habitats, even if their abundances are increasing.

In line with what we expected, we found that the rate of change in abundance with increasing occupancy is becoming significantly more rapid for neotropical migrants over time, but significantly less rapid for permanent residents. If the slope of the abundance–occupancy relationship continues to decline to 0 for the permanent residents, there will be no statistical relationship between abundance and occupancy for them. Short‐distance migrants show a nonsignificant decline in rate of change in abundance with occupancy, whereas the group comprised of all birds demonstrates a nonsignificant increase in slope of the abundance–occupancy relationship over time (driven largely by the long‐distance migrants).

There are a few phenomena that might explain these differences in change of abundance with occupancy rate across the migratory guilds. It is certain that for abundance–occupancy relationships to change significantly for different groups of animals, either the abundances must be differentially changing, or the occupancies must differentially change, or both. We have good reason to expect that occupancies would be different for at least the neotropical migrants, given their dispersal abilities (we treat this possibility below).

We considered whether permanent resident abundances have been decreasing over time, more than neotropical migrants' abundances. This hypothesis would make sense if permanent residents are faring worse on their wintering grounds (due to e.g., high snow cover, Doherty & Grubb, 2002) than are neotropical migrants. Conversely, we can imagine a scenario where breeding abundances are lower for neotropical migrants compared with permanent residents, due to loss of individuals from mortality during migration (Klaassen et al., 2014; Newton, 2006, 2007; Sillet & Holmes, 2002; Strandberg et al., 2009), or lowered reproductive output from low‐quality wintering habitat (Norris, Marra, Kyser, Sherry, & Ratcliffe, 2004). From Figure 4 (and associated *t* test), the hypothesis that permanent resident and neotropical migrant abundances differ is not supported.

If abundances of permanent residents and neotropical migrants do not differ, then perhaps occupancies for these groups differ. One possible mechanism for different occupancies is an earlier return to breeding grounds in recent years, resulting in stronger competition (by migrants) for breeding territories (and thus, larger occupancy for migrants). With continuing climate change, migratory distance is decreasing for at least some species (Curley, Manne, & Veit, 2019; Potvin, Välimäki, & Lehikoinen, 2016; Visser, Perdeck, Balen, & Both, 2009) as breeding and wintering ranges differentially shift. Further, with climate change‐associated phenology changes, migratory birds are returning earlier to breeding grounds (Cotton, 2003; Sparks et al., 2005). Species that have faster flight speeds have been shown to respond to changing climate with appropriate (earlier) phenological changes (Hurlbert & Liang, 2012). When we examined occupancy for neotropical migrants and permanent residents, we did find that neotropical migrants consistently have higher occupancy than permanent residents (Figure 5) and that this result is consistent over time.

**Figure 6:**
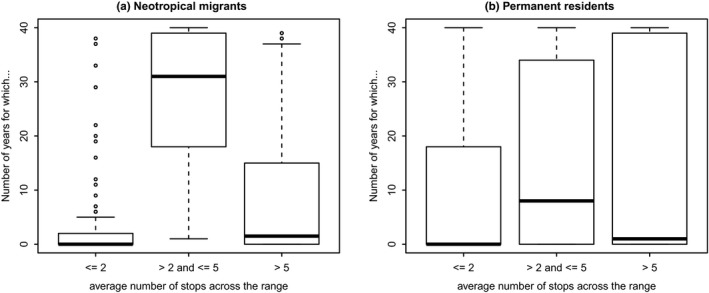
Permanent residents are more likely (than neotropical migrants) to be detected few times (average of 1 or 2 stops/route in a 50‐stop route) and on more than 5 stops (average > 5 stops/route), while neotropical migrants are more likely to have 2–5 average stops/route. See text for more explanation

It is possible that another, non‐climate‐related mechanism might explain the greater occupancy of neotropical migrants. The considerably larger vagility of neotropical migrants may correlate with greater range sizes (Böhning‐Gaese, Caprano, Ewijk, & Veith, 2006), and hence, greater occupancies via increased traversal (wandering) of the landscape, with consequent greater detections. However, other researchers have noted that analyses looking for a positive relationship between range size and dispersal ability have found conflicting results (Lester, Ruttenberg, Gaines, & Kinlan, 2007). We asked whether neotropical migrants have greater numbers of detections per transect than permanent residents. Out of 50 stops per BBS route, per species, and per year, we averaged the observed numbers of stops on which a species was detected in that year. We created bins of average numbers of stops (1–2 stops [1%–4% of stops], 3–5 stops [5%–10% of stops], >5 stops [>10% of stops]), and tallied the number of species‐year combinations that fell into these bins (Figure 6). *t* Tests of these data reveal that permanent residents have higher numbers of species‐year combinations with average number of stops ≤2 (*p* = .001), neotropical migrants have higher numbers of species‐year combinations with average number of stops >2 and <5 (*p* = 4.38 × 10^–7^), and permanent residents have higher numbers of species‐year combinations with average number of stops >5 (*p* = .02). Thus, permanent residents are more likely to have the lowest numbers of detections and also the highest numbers of detections, while neotropical migrants are more likely to have detections in the 5%–10% (of detections) range. We cannot count this as complete support for the idea that neotropical migrants are moving about the landscape more and thus being detected more often than are permanent residents.

Migration speed might explain why some neotropical migrants can arrive more quickly to breeding grounds and thus compete more effectively for breeding territories with resident birds. However, this argument does not work for short‐distance migrants, who should reach breeding grounds faster (even) than long‐distance migrants (Butler, 2003), but in this analysis did not show a significant change in rate of abundance increase with occupancy increase.

**Figure 7:**
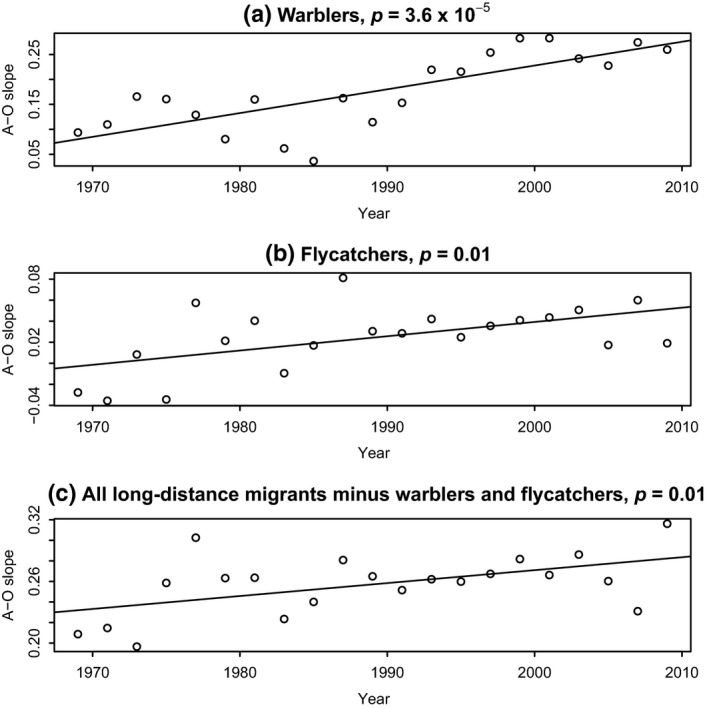
Within the neotropical migrants, a single cohesive group of species is not driving the increase in abundance–occupancy slope through time. (a) Warblers (*n* = 40); (b) Flycatchers (*n* = 16); (c) All long‐distance (neotropical) migrants excluding warblers and flycatchers

Last, we checked for the possibility of one large and cohesive species group within the long‐distance migrants driving the results. Within the long‐distance migrants, there are 40 wood warblers and 16 flycatcher species. For each of these groups and for the long‐distance group comprised of all species but flycatchers and wood warblers, we repeated the abundance–occupancy analyses. All three species groups demonstrate a significantly increasing slope of abundance–occupancy relationship through time (Figure 7).

## 5 CONCLUSIONS

The consistency of the abundance–occupancy relationship across many species groups and regions underscores its importance as a macroecological rule and governing principle of communities. It is important to understand the circumstances under which this rule will be altered (Fisher & Frank, 2004; Webb et al., 2007). [Here, if the decline of the slope of the abundance–occupancy relationship for permanent residents is not halted, the abundance–occupancy relationship for these species will cease to exist.] In these analyses, we have documented changing trends in abundance–occupancy for different groups of species, over a relatively long time period for ecological studies. This is one of only a few studies (that we know of) to examine abundance–occupancy relationships over time. Further, we have noted that some species show an increasing rate of abundance change with occupancy change and some species have a decreasing rate of abundance change with occupancy change. We have narrowed down the mechanism for this difference between species groups to a process impacting occupancy and have eliminated both better dispersal ability of the neotropical migrants and taxonomic sampling phenomena (one species group driving the relationship) as proximal causes.

Clearly, a temporally unvarying abundance–occupancy relationship is not universal. Human impacts on natural habitats and on global climate are unrelenting; we speculate that some of the variability in abundance–occupancy relationships observed here is due to human impacts on habitats. The role of habitat quality on community structure (Ficetola & De Bernardi, 2004; Hylander, Nilsson, Gunnar Jonsson, & Göthner, 2005) and resilience (Hughes et al., 2003) cannot be overstated. The next step in this line of research is to investigate how changing habitat quality/fragmentation may correlate with occupancy dynamics for North American bird species.

## CONFLICT OF INTEREST

None declared.

## AUTHORS' CONTRIBUTIONS

LM conceived the idea and carried out analyses. LM and RV designed the methodology. LM and RV wrote the manuscript.

## Data Availability

Breeding Bird Survey data available from the BBS‐USGS Patuxent Wildlife Research Center, https://www.pwrc.usgs.gov/bbs/ (Pardieck et al., 2016).
