# Supplementary material for: Temporal changes in abundance–occupancy relationships over 40 years
Source: Ecol Evol. 2019 Sep 30;10(2):602–11. doi: 10.1002/ece3.5505 (PMC6988556; doi:10.1002/ece3.5505)
Supplement: Supplementary file 1 [file ECE3-10-602-s001.docx]

Appendix S1: Partial autocorrelation for slopes of Abundance-occupancy relationships for a) Neotropical migrants, b) Short-distance migrants and c) Permanent residents

| **a) Neotropical migrants**  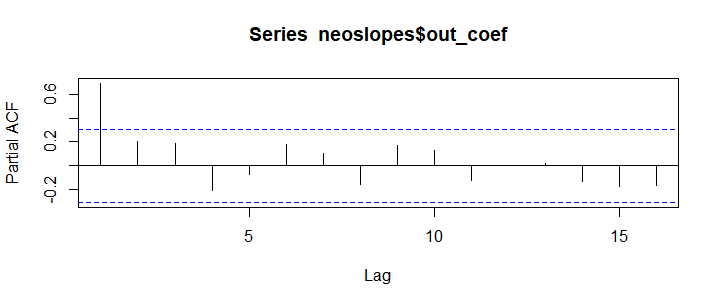 |
| --- |
|  |
| **b) Short-distance migrants**  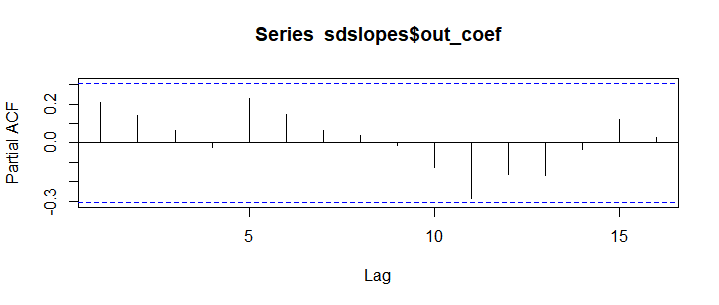 |
|  |
| **c) Permanent residents**  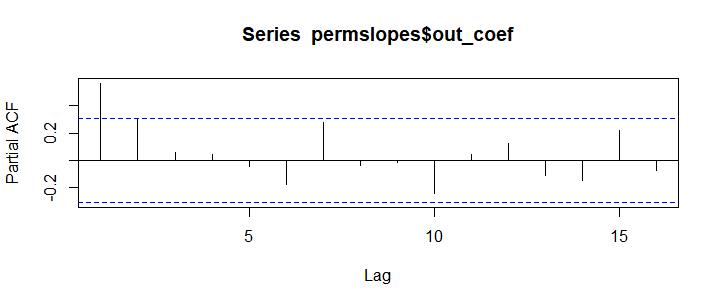 |
